# Elevated soluble LOX-1 predicts risk of first-time myocardial infarction

**DOI:** 10.1080/07853890.2023.2296552

**Published:** 2023-12-22

**Authors:** Alexandru Schiopu, Harry Björkbacka, Gayathri Narasimhan, Bi Juin Loong, Gunnar Engström, Olle Melander, Marju Orho-Melander, Jan Nilsson

**Affiliations:** a Department of Clinical Sciences Malmö, Lund University, Sweden; b Department of Transitional Science, Lund University, Sweden

**Keywords:** LOX-1, myocardial infarction, risk prediction, population cohort, disease mechanisms

## Abstract

**Background:**

There is an unmet clinical need for novel therapies addressing the residual risk in patients receiving guideline preventive therapy for coronary heart disease. Experimental studies have identified a pro-atherogenic role of the oxidized LDL receptor LOX-1. We investigated the association between circulating soluble LOX-1 (sLOX-1) and the risk for development of myocardial infarction.

**Methods:**

The study subjects (*n* = 4658) were part of the Malmö Diet and Cancer study. The baseline investigation was carried out 1991-1994 and the incidence of cardiovascular events monitored through national registers during a of 19.5 ± 4.9 years follow-up. sLOX-1 and other biomarkers were analyzed by proximity extension assay and ELISA in baseline plasma.

**Results:**

Subjects in the highest tertile of sLOX-1 had an increased risk of myocardial infarction (hazard ratio (95% CI) 1.76 (1.40-2.21) as compared with those in the lowest tertile. The presence of cardiovascular risk factors was related to elevated sLOX-1, but the association between sLOX-1 and risk of myocardial infarction remained significant when adjusting for risk factors.

**Conclusions:**

In this prospective population study we found an association between elevated sLOX-1, the presence of carotid disease and the risk for first-time myocardial infarction. Taken together with previous experimental findings of a pro-atherogenic role of LOX-1, this observation supports LOX-1 inhibition as a possible target for prevention of myocardial infarction.

## KEY MESSAGES

Activation of the lectin-like oxidized low-density lipoprotein receptor-1 (LOX-1) represents a possible target for treatment of the residual inflammatory risk in cardiovascular patients on guideline therapy.

Having high levels of soluble LOX-1, a marker of cellular LOX-1 activation, is associated with an increased risk for development of myocardial infarction and heart failure.

sLOX-1 levels correlated with major cardiovascular risk factors and biomarkers reflecting LDL oxidation.

Despite the introduction of several effective therapies for prevention of cardiovascular disease (CVD), such as high-dose statins, there persists an unacceptably high residual risk insufficiently targeted by currently available treatments [1,2]. Mechanisms responsible for this residual risk have been proposed to include reduced shear stress over established plaques promoting local inflammation and endothelial dysfunction, metabolic factors associated with pre- and established diabetes, and autoimmune responses against modified plaque antigens such as oxidized LDL [1,3,4]. A common feature of these proposed mechanisms is that they promote vascular inflammation. Support for the notion that inflammation represents a target for treatment of the residual CVD risk has recently come from clinical trials such as CANTOS, COLCOT and LoDoCo-2 [5–7]. However, a limitation with systemic anti-inflammatory therapies is that they are associated with an increased risk of infections [5], arguing that it is preferable to develop therapies more specific for vascular inflammation. The lectin-like oxidized low-density lipoprotein receptor-1 (LOX-1) has emerged as an interesting possible target for this [8–10]. LOX-1 is expressed primarily by endothelial cells but is also present on macrophages and other types of immune cells. It acts as a scavenger receptor for oxidized LDL and modified dysfunctional HDL, but it also triggers signaling pathways involved in inflammation, oxidative stress, and apoptosis, suggesting a possible role in atherosclerosis. Accordingly, endothelial overexpression of LOX-1 increases vascular inflammation and development of atherosclerosis in *ApoE^-/-^* mice [11], while the opposite effect is seen in atherosclerotic mouse models with a genetic deletion of LOX-1 [12,13]. Since LOX-1 activation promotes endothelial dysfunction and apoptosis, it also represents an interesting intervention target for endothelial erosions, a phenomenon that is responsible for as much as one third of all acute coronary events [14].

LOX-1 also exists in a circulating, soluble form termed sLOX-1. Oxidized LDL, as well as pro-inflammatory cytokines such as TNF-α, has been shown to induce shedding of sLOX-1 from cells [15] suggesting that the circulating level of sLOX-1 may reflect the level of membrane-bound LOX-1 activation as well as the general level of inflammation. sLOX-1 levels increase during acute myocardial infarction and have a diagnostic sensitivity and specificity equal to that of troponin T [16]. Interestingly, elevated sLOX-1, but not troponin T, was reported to be related to a higher frequency of plaque rupture in acute myocardial infarction [16]. Elevated levels of sLOX-1 have also been associated with increased risk of acute CVD events in subjects with stable coronary disease [17]. There is less information regarding the association between sLOX-1 and risk of myocardial infarction in the general population. Inoue and coworkers found no association between sLOX-1 and risk of myocardial infarction in a population cohort involving 2295 subjects aged 30-79 years, whereas an association of borderline significance was observed using an index including both sLOX-1 and LOX-1 ligands containing apolipoprotein B [18]. The possibility to reduce cardiovascular risk by inhibiting LOX-1 activation is presently investigated in a phase II trial (NCT NCT04610892). To investigate the possible role of LOX-1 in CVD we analyzed the association between baseline levels of sLOX-1, a marker of LOX-1 activation, and risk for development of myocardial infarction and heart failure in a population cohort of 4658 subjects aged 52-62 years followed for a mean of 19.5 years.

## Material and methods

### Population study cohort

The Malmö Diet and Cancer (MDC) study is a prospective population-based cohort (*n* = 28,449) study examining the association between diet and cancer [19]. Subjects born between 1926 and 1945 living in Malmö, Sweden were eligible for inclusion in the study. Between October 1991 and February 1994, every other participant was also invited to take part in a sub-study focusing on the epidemiology of carotid artery disease (MDC study cardiovascular cohort, *n* = 6,103) [20]. Out of these, 5405 came to a second baseline examination where fasting plasma samples were collected. We excluded 545 of these subjects from the present study due to incomplete clinical data, 118 subjects were further excluded because the analysis of their plasma samples did not pass the internal quality control for the biomarker analyses and 84 subjects because of previous myocardial infarction or heart failure. The remaining 4658 subjects were followed from baseline examination until first myocardial infarction or heart failure, emigration from Sweden or death, up until December 31^st^, 2014. Myocardial infarction was defined as a fatal or non-fatal myocardial infarction based on the International Classification of Diseases 9th and 10th revisions (ICD-9 and ICD-10) codes 410 and I21, and heart failure based on the corresponding codes 428 and I50, respectively. Diabetes at baseline was based on data from the Swedish National Diabetes Registry. Risk factors and carotid intima-media thickness were determined as previously described [20].

### Ethics

The study was approved by the Regional Ethical Review Board in Lund (LU 51-90) and was conducted in accordance with the Helsinki Declaration. All subjects gave written consent. The reporting of this cohort study is in accordance with the STROBE guidelines.

### Carotid ultrasound

B-mode ultrasonography (Acuson128 CT system, Siemens, Mountain View, Ca) of the right carotid artery was performed as previously described [21]. For the occurrence of plaques (defined as a focal thickening of the intima-media complex >1.2 mm and with an area ≥10 mm^2^) the bifurcation area of the right common carotid artery was scanned within a pre-defined section comprising 3 cm of the distal common carotid artery, the bifurcation, and 1 cm of the internal and external carotid artery.

### Release of sLOX-1 by cultured endothelial cells

Human umbilical vein endothelial cells (HUVECs) were grown in medium 200 containing 2% low serum growth supplement (Invitrogen) with addition of 1% penicillin-streptomycin (Gibco) in 24-well plates. Before stimulation, the cells were starved with 0.5% serum in medium 200 for 24 h. HUVECs were stimulated in starvation media with native LDL (60 μg/mL), copper-oxidized LDL (30 μg/mL), prepared as previously described [22], with or without the addition of LOX-1 antibody (5 μg/mL, Abcam) for 24 h in 37 °C incubator with 5% CO2. To assess sLOX-1 medium was stored in −20 °C and further analyzed using sLOX-1 human ELISA (Abcam).

#### Proximity extension assay

Plasma levels of sLOX-1, placental growth factor (PlGF), TRAIL receptor-2, caspase-8 and PECAM-1were analyzed by the Proximity Extension Assay (PEA) technique using the Proseek Multiplex CVD^96x96^ reagents kit (Olink Bioscience, Uppsala, Sweden) at the Clinical Biomarkers Facility, Science for Life Laboratory, Uppsala. The CV for intra-assay variation (within-run) and inter-assay variation (between-run) were 9% and 11% (sLOX-1), 12% and 13% (PlGF), 10% and 12% (TRAIL receptor-2), 5% and 10% (caspase-8) and 7% and 10% (PECAM-1), respectively and the analytical ranges 0.95-15625 pg/mL, 0.5-31250 pg/ml, 0.2-7812 ng/ml, 3.8-62500 pg/ml, and 0.95-15625 pg/ml, respectively. All data are presented as normalized protein expression values (NPX). General calibrator curves to calculate the approximate concentrations based on NPX as well as technical information about the assays are available on the Olink homepage (http://www.olink.com).

### Analyses of biomarkers reflecting LDL oxidation

Plasma oxidized LDL was measured by ELISA (Mercordia, Sweden) in a subset of 548 subjects. Lipoprotein-phospholipase A2 (Lp-PLA2) activity was measured using tritium-labelled platelet activating factor as substrate [23]. Measurement of autoantibodies against apolipoprotein B-100 peptide sequences by custom made ELISA was performed as previously described [24,25].

### Statistical analyses

Analysis of skewness and kurtosis were used to test for normality. Differences between means of normally distributed continuous variables were assessed with independent sample *t* tests and between skewed variables with the Mann-Whitney U-test. χ^2^ test was used for categorical variables. Correlation coefficients between continuous variables were calculated using the Spearman Rank test. A sparse Gaussian graphical model was computed from Spearman correlations with the graphical lasso algorithm [26] and the qgraph package in R v. 4.1.2. Differences between groups in the cell culture experiments were analyzed using Kruskal-Wallis test followed by the Benjamini, Krieger and Yekutieli false discovery rate test. The relation between sLOX-1 tertiles and a first cardiovascular event during follow-up was assessed by Kaplan Meier survival curves and quantified by Log rank test. Cox proportional hazards regression models were used to assess the hazard ratio (HR), and 95% confidence interval (CI) of first event in relation to sLOX-1 tertiles. The time variables used in Kaplan Meier curves and the Cox regressions models were time from the baseline investigation to first clinical event or end of follow up which was 2014-12-31. IBM SPSS Statistics 22 and GraphPad Prism 9 were used for statistical analysis.

### Role of the funder/sponsor

The funders had no role in the design and conduct of the study; collection, management, analysis, and interpretation of the data; preparation, review, or approval of the manuscript; and decision to submit the manuscript for publication.

## Results

In this prospective population-based study with 19.5 ± 4.9 years follow-up period, 440 subjects suffered a myocardial infarction (Table 1). All major cardiovascular risk factors, including elevated hsCRP, were overrepresented in the myocardial infarction group. The analysis of incident heart failure was restricted to those that developed heart failure unrelated to a myocardial infarction (*n* = 189), while those that developed heart failure in association with myocardial infarction (*n* = 36) were excluded from this analysis. Except for smoking and LDL cholesterol there was an over-representation of cardiovascular risk factors also in the heart failure group.

**Table 1.** Baseline clinical characteristics of study subjects with and without incident myocardial infarction and heart failure.

	No myocardial infarction (*n* = 4218)	Incident myocardial infarction (*n* = 440)	No incident heart failure (*n* = 4432)	Incident heart failure (*n* = 189)
Age (years)	57.2 ± 5.9	59.8 ± 5.5***	57.2 ± 5.9	61.1 ± 5.0***
Sex (n (%) males)	1575 (37.3)	254 (57.7)***	1712 (38.6)	96 (50.8)***
Current smoking (n (%))	890 (21.1)	113 (25.7)*	951 (21.5)	44 (23.3)
Diabetes (n (%))	143 (3.4)	42 (9.5)***	161 (3.6)	19 (10.1)***
BMI (kg/m^2^)	25.5 ± 3.9	26.6 ± 4.2***	25.5 ± 3.8	27.8 ± 5.2***
fB-glucose (mmol/L)	5.08 ± 1.18	5.57 ± 1.97***	5.10 ± 1.21	5.62 ± 1.86***
HbA1c (%)	4.86 ± 0.66	5.12 ± 1.05***	4.86 ± 0.69	5.18 ± 0.92***
LDL-C (mmol/L)	4.15 ± 0.98	4.35 ± 0.91***	4.17 ± 0.98	4.14 ± 0.97
HDL-C (mmol/L)	1.41 ± 0.37	1.26 ± 0.35***	1.40 ± 0.37	1.32 ± 0.36*
Triglycerides (mmol/L)	1.13 (0.85-1.55)	1.30 (0.96-1.80)***	1.14 (0.85-1.56)	1.21 (0.91-1.69)*
Systolic BP (mm Hg)	140 ± 19	149 ± 19***	140 ± 19	150 ± 21***
Diastolic BP (mm Hg)	86 ± 9	90 ± 10***	86 ± 9	90 ± 9***
hsCRP (mg/L)	1.30 (0.60-2.70)	1.70 (0.90-3.30)***	1.30 (0.60-2.70)	2.00 (1.00-4.40)***
LOX-1 (NPX)	4.09 ± 0.63	4.22 ± 0.65***	4.10 ± 0.63	4.20 ± 0.71*
TRAIL-R2 (NPX)	1.28 ± 0.44	1.38 ± 0.44***	1.28 ± 0.44	1.43 ± 0.41***
Caspace-8 (NPX)	1.52 ± 0.70	1.79 ± 0.77***	1.54 ± 0.70	1.57 ± 0.80
PECAM-1 (NPX)	6.30 ± 0.52	6.36 ± 0.52*	6.31 ± 0.52	6.28 ± 0.50
PlGF (NPX)	6.99 ± 0.42	7.11 ± 0.4***	7.00 ± 0.42	7.09 ± 0.43**

Variables with normal distribution are shown as mean and standard deviation and statistical differences between groups calculated by Student’s t-test. Variables with skewed distribution are shown as median and interquartile range and statistical differences between groups calculated by Mann-Whitney U-test. Statistical differences between categorical variables were analyzed by chi-square test. BMI; body mass index; f-glucose; fasting glucose; HbA1c; hemoglobin A1c, LDL-C; low-density lipoprotein cholesterol; HDL-C; high-density lipoprotein cholesterol; hsCRP; high-sensitivity C-reactive protein. Diabetes included both type 1 and type 2. *p < 0.05, **p < 0.005 and ***p < 0.001 as compared with the respective group without event. Thirty-six subjects that developed heart failure after suffering myocardial infarction were excluded from the analyses of incident heart failure.

### sLOX-1 and incidence of myocardial infarction

**Figure 1.**
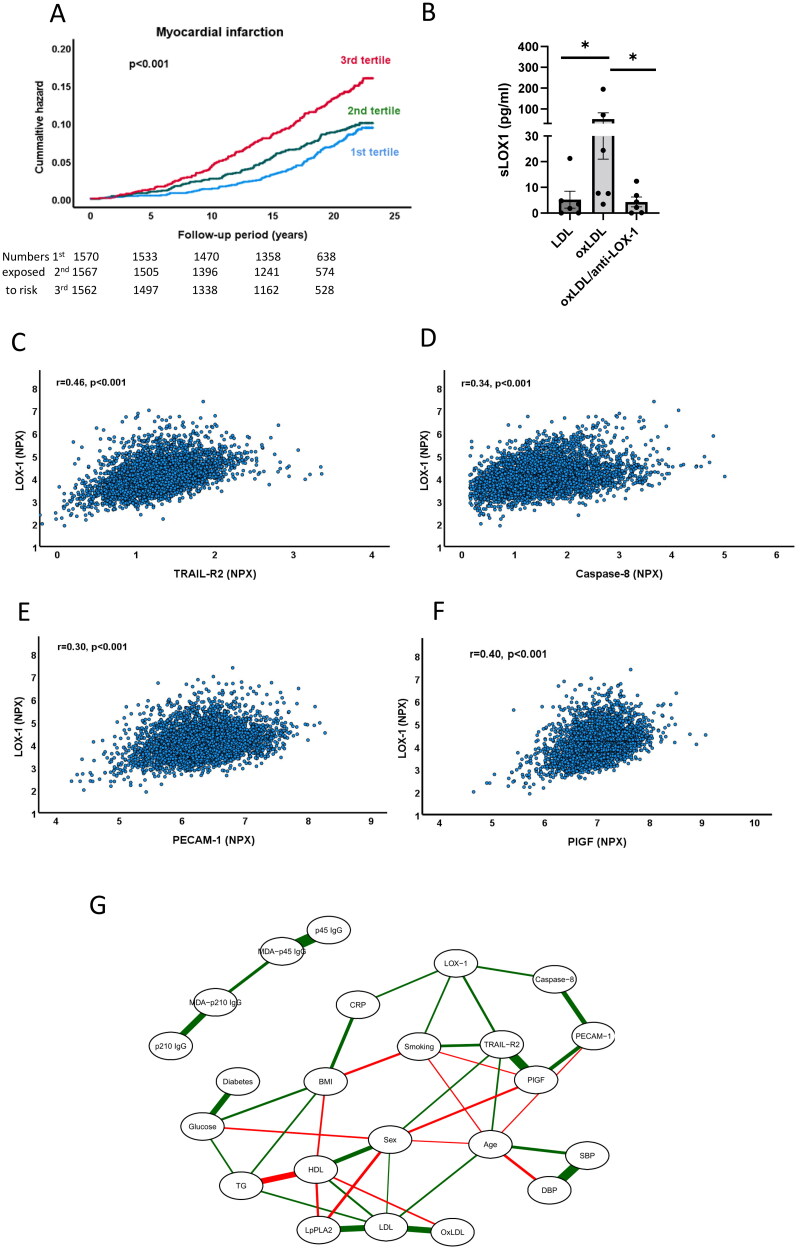
Associations of sLOX-1 with incident myocardial infarction and markers of endothelial stress. (A) Kaplan-Meier plot showing the association between sLOX-1 tertiles and incidence of myocardial infarction. Log rank test was performed to calculate the P value. (B) Release of sLOX-1 from cultured HUVECs exposed to LDL (63 µg/mL) or oxidized LDL (34 µg/mL) with or without anti-LOX-1 IgG (5 µg/mL) for 24 h. Differences between groups were analyzed using Kruskal-Wallis test followed by the Benjamini, Krieger and Yekutieli false discovery rate test. *p < 0.05. (C-F) Scatter plots demonstrating associations between sLOX-1 and plasma markers of endothelial stress in the study cohort. Correlation coefficients were calculated using the Spearman rank test. (G) Computed sparse Gaussian graphical model showing partial Spearman correlations with (*r* > 0.1) between markers of cellular stress and baseline characteristics in Table 1.

Baseline sLOX-1 was elevated in subjects with incident myocardial infarction (Table 1). As compared with the lowest tertile of sLOX-1, those in the highest tertile had a 77% higher risk of myocardial infarction (Figure 1(A) and Table 2 (regression model 1)). Except for male sex and high BMI, sLOX-1 levels demonstrated significant association with all major cardiovascular risk factors (Table 3). Notably, smoking was almost three times as frequent among those in the highest tertile as compared with those in the lowest. After adjusting for the risk factors included in the Framingham risk score as well as for diabetes, the association between sLOX-1 and myocardial infarction was weakened but remained statistically significant (Table 2).

**Table 2.** Cox proportional hazard regression models of LOX-1 tertiles, myocardial infarction and heart failure.

	1^st^ tertile (*n* = 1580)	2^nd^ tertile (*n* = 1581)	3^rd^ tertile (*n* = 1581)
Myocardial infarction, n	121	128	191
M1: HR (95%CI)	1	1.14 (0.89-1.46)	1.76 (1.40-2.21)***
M2: HR (95%CI)	1	1.02 (0.79-1.30)	1.56 (1.24-1.96)***
M3: HR (95%CI)	1	0.92(0.72-1.17)	1.27 (1.01-1.60)*
Heart failure, n	63	49	77
M1: HR (95%CI)	1	0.83 (0.57-1.21)	1.35 (0.97-1.88)
M2: HR (95%CI)	1	0.71 (0.49-1.03)	1.15 (0.82-1.60)
M3: HR (95%CI)	1	0.65 (0.45-0.95)*	1.00 (0.71-1.41)

Cox proportional hazard regression models (M) were adjusted as follows: M1 was unadjusted, M2 adjusted for age, sex and M3 was adjusted for age, sex, total and HDL cholesterol, systolic blood pressure, diabetes and current smoking. *p < 0.05 and ***p < 0.001 vs 1^st^ tertile. Thirty-six subjects that developed heart failure after suffering myocardial infarction were excluded from the analyses of incident heart failure. 1^st^ tertile 1.92-3.81 NPX, 2^nd^ tertile 3.81-4.28 NPX and 3^rd^ tertile 4.48-7.39 NPX.

**Table 3.** Associations between LOX-1 tertiles, cardiovascular risk factors and biomarkers related to oxidized LDL and endothelial stress.

	LOX-1			
	1^st^ tertile (*n* = 1552)	2n tertile (*n* = 1553)	3^rd^ tertile (*n* = 1552)	P linear trend
***Cardiovascular risk factors***				
Age (years)	56.6 ± 6.0	57.8 ± 6.0	57.9 ± 5.9	<0.001
Sex (% males)	38.2	38.8	40.8	ns
Current smoking (%)	11.9	20.7	32.1	<0.001
Diabetes (%)	3.0	4.2	4.7	0.01
BMI (kg/m^2^)	25.5 ± 3.8	25.5 ± 3.7	25.7 ± 4.	ns
fB-glucose (mmol/L)	5.02 ± 1.01	5.15 ± 1.40	5.21 ± 1.39	<0.001
LDL (mmol/L)	4.04 ± 0.95	4.17 ± 0.98	4.29 ± 0.99	<0.001
HDL (mmol/L)	1.44 ± 0.38	1.39 ± 0.37	1.34 ± 0.36	<0.001
TG (mmol/L)	1.04 (0.79-1.45)	1.16 (0.87-1.61	1.23 (0.93-1.67)	<0.001
Systolic BP (mm Hg	140 ± 18	141 ± 19	142 ± 19	<0.001
Diastolic BP (mm Hg)	86 ± 9	87 ± 9	87 ± 10	0.001
hsCRP (mmol/L)	1.00 (0.60-2.00)	1.30 (0.50-2.60)	1.80 (0.90-3.70)	<0.001
***Biomarkers related to oxidized LDL***				
Oxidized LDL (U/L)	117 ± 33	120 ± 29	126 ± 36	0.01
LpPLA2 activity (nmol/min/mL)	43.6 ± 12.4	45.1 ± 12.6	47.1 ± 13.5	<0.001
ApoB p45 IgG (Abs)	0.22 (0.09-0.45)	0.23 (0.11-0.46)	0.24 (0.11-0.47)	ns
MDA-apoB p45 IgG (Abs)	0.35 (0.23-0.55)	0.36 (0.25-0.55)	0.38 (0.24-0.58)	ns
ApoB p210 IgG (Abs)	0.44 ± 0.23	0.43 ± 0.23	0.42 ± 0.23	0.003
MDA-apoB p210 IgG (Abs)	0.69 ± 0.30	0.69 ± 0.34	0.70 ± 0.32	ns
***Biomarkers related to endothelial stress***				
TRAIL receptor-2 (NPX)	1.07 ± 0.39	1.33 ± 0.37	1.49 ± 0.44	<0.001
Caspace-8 (NPX)	1.26 ± 0.63	1.57 ± 0.68	1.80 ± 0.70	<0.001
PECAM-1 (NPX)	6.11 ± 0.51	6.35 ± 0.46	6.47 ± 0.51	<0.001
PlGF (NPX)	6.78 ± 0.40	7.06 ± 0.34	7.17 ± 0.40	<0.001

Tertiles of sLOX-1 with 1^st^ tertile being the lowest tertile. BMI; body mass index, LDL; low-density lipoprotein, HDL; high-density lipoprotein, SBP; systolic blood pressure, CRP; C-reactive protein, ns; not significant. Diabetes included both type1 and type 2. Linear trends across tertiles were calculated with one-way ANOVA for continuous normally distributed variables (presented as mean ± SD), Kruska-Wallis one-way ANOVA test for continuous non-normally distributed variables (presented as median with IQR) and χ^2^ test for categorical variables. Oxidized LDL was only determined in a randomly selected subgroup (*n* = 548).

Oxidized LDL is considered a key ligand for LOX-1 [9], but this notion is based mainly on studies performed on cultured cells. We first investigated if the release of sLOX-1 from cells exposed to oxidized LDL is dependent on activation of the LOX-1 receptor and found that treatment of cultured human umbilical vein endothelial cells with a LOX-1 blocking antibody fully inhibited the release of sLOX-1 induced by oxidized LDL (Figure 1(B)). To obtain clinical evidence for a role of oxidized LDL in LOX-1 activation we next analyzed the association of sLOX-1 with biomarkers reflecting oxidation of LDL. We found that high sLOX-1 was associated with increased levels of oxidized LDL and lipoprotein phospholipase A2 (LpPLA2, Table 3). LpPLA2 is an enzyme that cleaves oxidized fatty acids and that primarily is bound to LDL [27]. We also found an inverse association between sLOX-1 and IgG autoantibodies against the p210 sequence in apolipoprotein B-100 (Table 3). Subjects with high levels of these autoantibodies has previously been shown to have a lower risk of myocardial infarction [25]. These observations provide clinical support for an association between LDL oxidation and release of sLOX-1.

We next investigated the relations between sLOX-1 and biomarkers reflecting cellular stress. TRAIL receptor 2 is a so-called death receptor that is released from cells receiving an external signal to activate apoptosis [28,29]. Caspase-8 is a key apoptosis-signaling enzyme but is not released from cells until they begin to disintegrate [30]. Both TRAIL receptor-2 and caspase-8 in the circulation correlated significantly with sLOX-1 (Figure 1(C,D)). TRAIL receptor-2 and caspace-8 were elevated in subjects with incident myocardial infarction, but only TRAIL receptor-2 was associated with incident heart failure (Table 1). Moreover, sLOX-1 also correlated with the plasma level of the endothelial marker PECAM-1 (CD31) as well as with placental growth factor (PlGF, Figure 1(E,F), and Table 3). The latter is a growth factor for endothelial cells that stimulate growth of stressed cells through auto- and paracrine mechanisms [31]. Both biomarkers were also associated with incident myocardial infarction, while only PlGF was associated with incident heart failure (Table 1). Next, we explored relationships between markers of cellular stress and baseline characteristics in a Gaussian graphical model (Figure 1(G)). Soluble LOX-1 showed significant partial correlations, i.e. correlations when controlling for all other variables in the analysis, with Caspase-8, TRAIL receptor-2, smoking and hsCRP. Although PECAM-1 and PlGF did not display significant partial correlations to sLOX-1, they had respectively strong significant partial correlations to Caspase-8 and TRAIL receptor-2. Collectively, these observations indicate that sLOX-1 levels are associated with markers of endothelial stress.

### sLOX-1 and carotid disease

Subjects with a plaque in the carotid bifurcation at baseline (*n* = 1553) had higher levels of sLOX-1 (4.18 ± 0.67 versus 4.61 ± 0.62 NPX, *p* < 0.001). This difference remained significant at *p* < 0.001 when adjusting for age and sex in a logistic regression model, but lost significance when also adjusting for the remaining factors in the Framingham risk score and diabetes.

### sLOX-1 and heart failure

**Figure 2.**
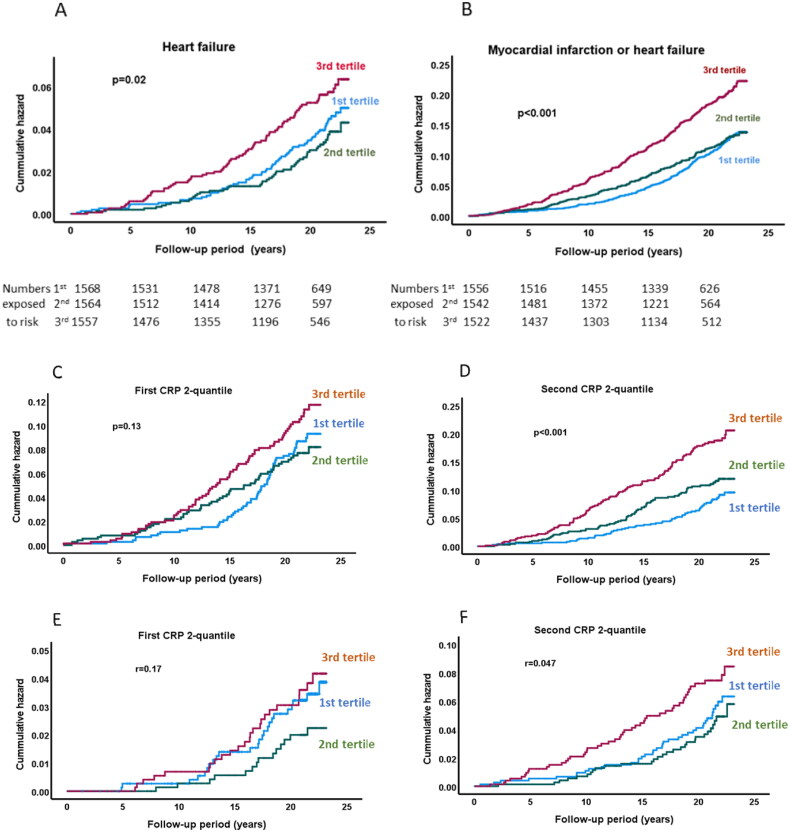
Associations of sLOX-1 with incident heart failure and cardiovascular events in subjects with below or above hsCRP. Kaplan-Meier plot showing the association between sLOX-1 tertiles and (A) incidence of heart failure, and myocardial and heart failure combined (B). Log rank test was performed to calculate the P values. Kaplan-Meier plots showing the association between sLOX-1 tertiles and incidence of myocardial infarction in subjects with (C) below median or (D) above median hsCRP at baseline. E and F show the association between sLOX-1 tertiles and incidence of heart failure in subjects with below median or above median hsCRP at baseline. Log rank test was performed to calculate the P values.

Experimental studies have implicated LOX-1 in cardiac fibrosis and heart failure [32,33]. To explore possible clinical evidence for a role of LOX-1 in heart failure we compared baseline sLOX-1 levels in subjects that did not have an incident myocardial infarction but developed heart failure during follow-up (*n* = 189) with those who did not (*n* = 4432). Except for smoking and high LDL cholesterol, all cardiovascular risk factors were more common among subjects with incident heart failure (Table 1). Baseline sLOX-1 was also higher in the heart failure group (Table 1), but this association was weaker than for myocardial infarction (Figure 2(A)) and was lost after adjusting Framingham risk factors (Table 2). A Kaplan-Meier plot based on the combined incident myocardial infarction and heart failure endpoints is Figure 2(B).

### Influence of hsCRP on the associations of sLOX-1 with cardiovascular events

To investigate to what extent the association between sLOX-1 and the risk of myocardial infarction and heart failure was dependent on the hsCRP level we analyzed subjects with hsCRP below or above median at baseline. The results showed that the association of sLOX-1 with increased risk of both myocardial infarction and heart failure was restricted to those with above median hsCRP (Figure 2(C**–**F)).

## Discussion

In the present study we found an association between high circulating sLOX-1 and an increased risk of first-time myocardial infarction. Plasma sLOX-1 levels correlated significantly with several cardiovascular risk factors and when adjusting for the factors included in the Framingham risk score, as well as for diabetes, the association between sLOX-1 and risk of myocardial infarction became weaker. A likely interpretation of these observations is that the risk factors directly or indirectly activate the release of sLOX-1 from cells. Oxidized LDL is a key ligand for LOX-1 and experimental studies have shown that oxidized LDL increase the release of sLOX-1 from endothelial cells [9,10,15]. In the present study we found that oxidized LDL stimulates the release of sLOX-1 through activation of the LOX-1 receptor and that subjects with high levels of LDL and oxidized LDL have increased plasma sLOX-1. Moreover, high plasma levels of LP-PLA2, an enzyme involved in the removal of oxidized fatty acids from LDL [27], was also associated with increased sLOX-1. Also, metabolic risk factors characteristic for diabetes and pre-diabetes such as high glucose, triglycerides and low HDL were associated with increased sLOX-1. There is less data from experimental studies to explain these associations. However, the metabolic changes in diabetes are also associated with an increased generation of oxidized LDL [34,35] and dysfunctional HDL [36,37] that may interact directly with the LOX-1 receptor.

There is evidence from experimental animal models that activation of LOX-1 promotes the development of atherosclerosis [11–13]. Against this background, our findings could be interpreted to support the notion that activation of LOX-1 by oxidized LDL and other ligands contributes to progression of atherosclerosis and therefore increases the risk of myocardial infarction. If this is correct, inhibition of oxidized LDL/LOX-1 interaction represents an interesting possibility for treatment of patients considered to have a residual cardiovascular risk. As compared to systemic anti-inflammatory therapy, this approach targets more specifically vascular inflammation and is less likely to be associated with the increased risk of infections observed in cardiovascular intervention trials using systemic anti-inflammatory therapy [5]. This concept is currently being tested in several clinical trials. A phase I clinical trial with an anti-LOX-1 neutralizing antibody (MEDI6570; NCT03654313) has been completed and a phase II trial (NCT NCT04610892) is currently being carried out to evaluate the effect of this antibody on markers of coronary disease and heart failure in 400 patients with a history of myocardial infarction and persistent inflammation. The GLACIER trial (Goal of Oxidized LDL and Activated Macrophage Inhibition by Exposure to a Recombinant Antibody) investigated the effect of treatment with an oxidized LDL-specific antibody on carotid plaque inflammation in 147 patients with stable CVD using positron emission tomography with 18 F-fluorodeoxyglucose at baseline and after 12 weeks [38]. The investigators were unable to detect any effect of treatment on plaque inflammation but speculated that this could be dependent on that the study subjects already were sufficiently stabilized by treatment with statins. A second phase II trial (NCT04776629) testing the effect of this antibody (Orticumab) on markers of inflammation and coronary disease in 75 subjects with severe psoriasis and cardiometabolic risk factors is ongoing. The outcome of these trials will provide important information regarding the role of oxidized LDL/LOX-1 activation in coronary disease and the potential benefit of targeting this pathway. However, there may exist alternative interpretations to the present finding of an association between elevated sLOX-1 and an increased risk of myocardial infarction. Studies performed on cultured cells have identified that pro-inflammatory cytokines can increase the release of sLOX-1 [15,39]. In line with this, our study found an association between hsCRP and sLOX-1. Accordingly, it cannot be excluded that inflammation independently drives both the release of sLOX-1 and progression of atherosclerosis. However, we also found that the association between sLOX-1 and risk of cardiovascular events was restricted to those with elevated hsCRP at baseline. This is suggestive of a link between markers of inflammation and sLOX-1 in cardiovascular disease. In this context it is interesting to note that reduction of cardiovascular risk by blocking IL-1β in the CANTOS study was restricted to those with elevated hsCRP at baseline [5].

Although several earlier studies have reported increased levels of sLOX-1 during acute myocardial infarction [16,40], only one previous study has specifically investigated if elevated sLOX-1 predicts risk of myocardial infarction in the general population. This study, which included 2437 Japanese men and women with a mean follow up of 11 years, did not find any association between baseline sLOX-1 and incident coronary events (myocardial infarction, coronary artery bypass surgery or angioplasty) [18]. However, only 68 incident cases of coronary events occurred during this study, suggesting that it may not have had sufficient power. Moreover, two recent studies using machine learning and proximity extension assay analysis of 368 [41] and 276 [42] potential cardiovascular biomarker proteins found no or only small independent predicted value of sLOX-1. In contrast, a previous study based on the Malmö Diet and Cancer cohort involving 4703 subjects with a mean follow up period of 16.6 years reported a 75% higher risk of ischemic stroke among those in the highest baseline tertile of sLOX-1 [15]. This report also included studies of atherosclerotic plaques obtained at surgery demonstrating that plaques with a high expression of LOX-1 contained more oxidized LDL and were more inflamed. The difference in results using machine learning to identify the best predictive markers in a larger set of potential biomarkers of cardiovascular risk and hypothesis-testing of single biomarkers are not necessarily in conflict with each other.

We did not see any clear association between baseline sLOX-1 and the risk of developing non-ischemic heart failure. These findings further support that the link between LOX-1 and CV risk is due to its relationship with coronary artery disease rather than direct effects on the cardiac muscle.

There are some limitations of the present study that should be considered. Biomarker analyses were only performed on baseline samples, and we lack information of variation in levels during follow-up, including the possible effects of medications prescribed after the baseline investigation. Although our study cohort was relatively large the results still need to be confirmed in other cohorts, particularly since unbiased proteomic studies have not identified sLOX-1 as one of the major markers of CVD risk [41,42]. Oxidized LDL was only determined in a randomly selected subgroup of 548 subjects and the analysis of the association with sLOX-1 did not have the same statistical power as for the other biomarkers. The presence of diabetes at baseline did not differentiate between type 1 and type 2. Most importantly, it should be kept in mind that the associations observed in the present study cannot be taken as proof of causality.

In conclusion this prospective population study with almost 20 years follow-up demonstrates an association between elevated sLOX-1 and risk for myocardial infarction as well as the presence of carotid disease. Taken together with previous experimental findings of a pro-atherogenic role of LOX-1, these observations support LOX-1 inhibition as a target for prevention of cardiovascular disease and suggest that this may be used to address the residual risk in patients receiving current state-of-the-art preventive treatment.

## Authors contributors

AS and JN designed the study, made statistical analyses, and wrote the first draft of the manuscript. GN and BJL performed cell culture studies and analyzed the results of these. HB performed network analysis. JN, OM, MOM and GE were responsible for biomarker analyses and study cohort. All authors made critical revision of the manuscript.

## Data Availability

The data underlying this article will be shared on reasonable request to the corresponding author.
